# Optimization of Bioethanol Production from Enzymatic Treatment of Argan Pulp Feedstock

**DOI:** 10.3390/molecules26092516

**Published:** 2021-04-26

**Authors:** Jihane Zeghlouli, Gwendoline Christophe, Amine Guendouz, Cherkaoui El Modafar, Abdeljalil Belkamel, Philippe Michaud, Cédric Delattre

**Affiliations:** 1 Laboratoire d’Agrobiotechnologie et Bioingénierie, Faculté des Sciences et Techniques Marrakech, Université Cadi Ayyad, Marrakesh 40000, Morocco; Jihane.ZEGHLOULI@etu.uca.fr (J.Z.); a.guendouz@uca.ac.ma (A.G.); elmodafar@uca.ac.ma (C.E.M.); belkameljalil@gmail.com (A.B.); 2 Institut Pascal, Université Clermont Auvergne, CNRS, Clermont Auvergne INP, F-63000 Clermont-Ferrand, France; gwendoline.christophe@uca.fr (G.C.); philippe.michaud@uca.fr (P.M.); 3 Institut Universitaire de France (IUF), 1 Rue Descartes, 75005 Paris, France

**Keywords:** argan, pulp, ethanol, cellulase, fermentation, cellulose

## Abstract

Argan pulp is an abundant byproduct from the argan oil process. It was investigated to study the feasibility of second-generation bioethanol production using, for the first time, enzymatic hydrolysis pretreatment. Argan pulp was subjected to an industrial grinding process before enzymatic hydrolysis using Viscozyme L and Celluclast 1.5 L, followed by fermentation of the resulting sugar solution by *Saccharomyces cerevisiae*. The argan pulp, as a biomass rich on carbohydrates, presented high saccharification yields (up to 91% and 88%) and an optimal ethanol bioconversion of 44.82% and 47.16% using 30 FBGU/g and 30 U/g of Viscozyme L and Celluclast 1.5 L, respectively, at 10%*_w_*_/*v*_ of argan biomass.

## 1. Introduction

By 2030, international energy demands are envisaged to increase by 53% [1]. Nowadays, fossil fuels are the world’s primary energy source with an estimated share of total final energy consumption of 79.9% [2]. However, the overexploitation of fossil energies has a negative impact on the health of the environment, which is suffering from pollution more than ever. Therefore, growing environmental concerns and current energy demands have urged the scientific community, governments, and companies to search for alternative energy sources to reduce dependence on petroleum and fight against climate change [3]. For these reasons, there is a rising interest in biofuels, in particular second-generation biofuels [4,5,6].

Bioethanol is actually the most produced biofuel, corresponding to about 59% of biofuel production in 2019 in terms of energy [1]. It represents a sufficient and ecofriendly alternative, taking into account that it can be used in gasoline engines and modified engines in high proportions. Furthermore, it contains a high amount of oxygen, which improves the combustion, and a high-octane number, which allows the operation of the engines even at higher compression [7,8]. Currently, commercially available bioethanol is exclusively of the first-generation (1 G) type, using agricultural products such as sugar beet in the European Union (EU), corn in the US, and sugarcane in Brazil [9,10,11]. One of the main disadvantages of 1 G bioethanol is the competition with food for arable lands, leading to serious concerns about socioeconomic and environmental consequences [9,10,11]. Otherwise, lignocellulosic is a potential candidate feedstock for the production of second-generation (2 G) bioethanol. It comprises different types of biomass, including all kinds of agricultural residues (e.g., wheat straw, corn stover, and sugarcane bagasse), energy crops (perennial grasses), and forest materials (principally woody materials), and it is indeed preferred as it is perceived as non-competitive with agri-food [10]. Also, it allows for low-cost biofuel production because the price of this biomass is estimated as being the lowest compared to starch, which is presently used to produce bioethanol [12].

The argan tree (*Argania spinosa* (L.) Skeels) is the second-most common forest species in Morocco after the holm oak and occupies about 871,210 hectares with a density estimate of 20 million trees and a production of 500 kg of fruit/ha/year [13]. In addition to its environmental qualities, it has an important economic and social role considering that 3.5 million local lives depend on this ecosystem [14]. This noble forest species is widely known for being the source of argan oil, which has numerous nutritional, therapeutic, and cosmetic benefits. As a consequence, the argan oil production process generates a significant number of byproducts (43% pulp, 52.6% shells, and 2% oil cake) that are hardly being exploited at present. The pulp, rich in carbohydrates and fibers, is the least-valorized byproduct; it is mainly used as input in small amounts in the diets of livestock [15,16].

An alternative way to valorize this agricultural byproduct would be through the production of 2 G bioethanol in order to develop a reliable and sustainable energy source for the arganery region in Morocco that would align well with energy transition policy and the promotion of a cleaner environment. This could have the advantage of promoting the valorization of local argan byproduct with a vision to set up an argan biorefinery.

The used feedstock influences the process of ethanol production. In general, lignocellulosic biomasses present a complex structure that can be broken down into fermentable sugars by enzymatic hydrolysis or chemically by sulfuric or other acids [17,18]. Acid hydrolysis is not a green process and it is restricted by the neutralization steps and the inhibitory byproduct production [17,19]. However, enzymatic hydrolysis is a green process, and it may prove useful due to the specificity of the enzymes. It can be considered as a critical step in the process of converting cellulose to glucose, which is carried out by cellulase enzymes under mild reaction conditions (pH 4.8–5.0 and temperature 45–50 °C). These conditions require less energy and they do not cause the formation of inhibitory compounds or equipment corrosion [20,21]. Most essentially, enzymatic hydrolysis procures high yields of sugars, 80–95%, and it is environmentally friendly [22]. Thus, the efficiency of enzymatic hydrolysis is influenced by optimized conditions, such temperature, time, pH, enzyme loading, and substrate concentration [23].

Up to now, and to the best of our knowledge, there has been only one reported study on the pretreatment of argan pulp using acid hydrolysis for bioethanol production [24], with not much information on fermentation and bioethanol production.

In this present work, the goal was, therefore, to hydrolyze and investigate the fermentation process of an industrially grounded and readily available argan pulp for use as an abundant and cheap lignocellulosic source of 2 G bioethanol. This process presents the simultaneous advantages of promoting the valorization of argan oil production and contributing to the economic development of local lives dependent on the arganery ecosystem. This study is primarily interested in the optimization of the green process of enzymatic hydrolysis of argan pulp using Celluclast (a commercial cellulolytic extract from *Trichoderma reesei*) and Viscozyme (a cell-wall-degrading extract from *Aspergillus* sp.) in order to obtain a high concentration of fermentable sugars. Subsequently, the feasibility of bioethanol production after fermentation of hydrolyzates by *Saccharomyces cerevisiae* was investigated.

## 2. Results and Discussion

### 2.1. Chemical Composition of Argan Pulp

Argan pulp is very rich in soluble carbohydrates. It is used primarily as an input in the diet of livestock. Several studies have been performed on the fruit of argan trees and argan oil composition [25,26,27,28,29], however, not much information is available on the chemical composition of the pulp. The majority of reports available on argan pulp were dedicated to its secondary metabolites, such as phenolic compounds [30], sterols, triterpenes [31,32], latex [33], and saponins [28], but no data on the pulp carbohydrates or cell wall fibers content are available.

#### 2.1.1. Main Chemical Constituents

The content of main chemical constituents of argan pulp is shown in Table 1. An antecedent qualitative study of Argan pulp highlighted that it contains 6% fat, 5.9% proteins, and 20% to 50% moisture [30]. Similarly, argan pulp dry matter, protein, and fat contents have been reported in nine different regions. Depending on the region, dry matter content ranged from 83.75% to 89.82%, while fat and protein content accounted for 5.37% to 7.40% and 5.01% to 9.26%, respectively [34].

#### 2.1.2. Fiber Content

The plant cell wall is mostly composed of carbohydrate fibers (cellulose and hemicelluloses), which are bound together with lignin and pectin, as well as proteins, enzymes, and phenolic components. Very few studies have focused on the argan pulp polysaccharides. The average content of cellulose (=ADF-lignin), NDF, ADF, hemicelluloses (=NDF-ADF), and lignin in argan pulp are summarized in Table 1.

The content of cellulose (19.35%) and hemicellulose (6.75%) in our samples was higher than those reported for argan pulp from nine different localities varying from 8.16% to 16.53% and 3.27% to 5.02% for cellulose and hemicelluloses, respectively [34]. These differences can be explained by the varying environmental conditions, such as light, temperature, and osmotic conditions, which can affect cellulose synthesis [35]. The chemical composition of argan pulp is mainly made up of cellulose, which is the simplest natural polymer to convert into its monomers and helps to improve the fermentation process, followed by hemicellulose and then lignin.

#### 2.1.3. Sugar and Phenolic Content

Sugar and phenolic content are presented in Table 1. The obtained total sugar (789.35 mg/g DW), reducing sugar (120.32 mg/g DW), and phenolic (76.17 mg EGA/g DW) contents were in line with the results highlighted by Zouhair, et al. (2020) [34] using the same procedures. Based on their results, the total sugar, reducing sugar, and phenolic compounds were 358.28–668.38 mg/g DW, 121.99–267.89 mg/g DW, and 74.37–101.24 mg EGA/g DW, respectively. Additionally, Zhar, et al. [36] reported that the argan pulp spherical morphotype is very rich in sugar (85.5–353.33 mg/g FW) and the phenolic content became significant with the maturation for all fruit morphotypes.

### 2.2. Argan Pulp Enzymatic Hydrolysis

**Figure 1:**
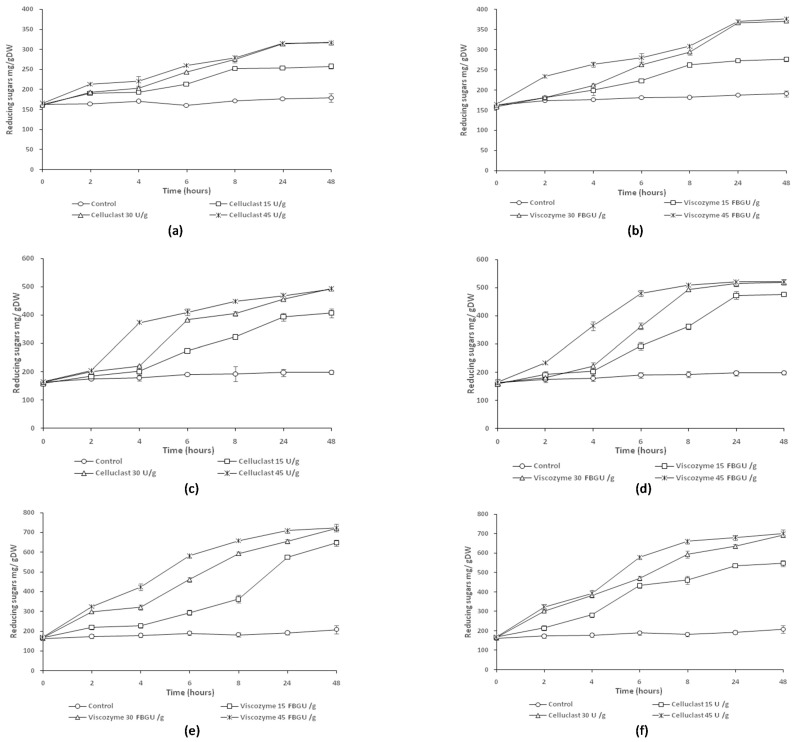
Analysis of enzymatic hydrolysis of argan pulp at three different substrate loadings (2%*_w_*_/*v*_, 5%*_w_*_/*v*_, and 10%*_w_*_/*v*_), using Celluclast and Viscozyme at different concentrations monitoring the reducing sugar concentrations. (**a**) Pulp at 2%*_w_*_/*v*_ using Celluclast, (**b**) pulp at 2%*_w_*_/*v*_ using Viscozyme, (**c**) pulp at 5%*_w_*_/*v*_ using Celluclast, (**d**) pulp at 5%*_w_*_/*v*_ using Viscozyme, (**e**) pulp at 10%*_w_*_/*v*_ using Celluclast, and (**f**) pulp at 10%*_w_*_/*v*_ using Viscozyme.

The efficiency of the enzymatic hydrolysis of argan pulp, the enzyme loading, and the biomass loading in the production of bioethanol under optimal conditions was studied. Figure 1 depicts the evolutions of reducing sugar concentrations over time for three different biomass loadings (2%*_w_*_/*v*_, 5%*_w_*_/*v*_, and 10%*_w_*_/*v*_), in the presence of Celluclast and Viscozyme at different concentrations (15 U/g, 30 U/g, and 45 U/g) and (15 FBGU/g, 30 FBGU/g, and 45 FBGU/g), respectively.

#### 2.2.1. Effect of Enzyme Loading

As shown in Figure 1, for both cellulosic extracts the increase in enzyme concentration led to an increase in reducing sugars released. Thus, argan pulp with a higher enzyme loading demonstrated a higher production of reducing sugars, which reached nearly 700.36 ± 0.8 and 723.36 ± 0.22 mg/g DW for Celluclast and Viscozyme, respectively, at a biomass loading of 2%*_w_*_/*v*_ (Figure 1a,b). The concentrations of reducing sugars released using the higher amount of enzyme (30 U/g of Celluclast or 30 FBGU/g of Viscozyme) at 24 h were approximatively the same after 48 h of enzymatic treatment. The hydrolysis efficiency was significantly increased at the beginning of the reaction and became stable after 24 h. The same conclusions were reported by Abdou Alio, et al. (2020) [37] after enzymatic hydrolysis on a wet pretreated sawdust mixture with both 50 and 70 FPU/g enzyme. Moreover, in another study reported by Guerrero, et al. [38], the bioethanol production from banana waste was studied at different enzyme concentrations (7.5–22.5 FPU/g^−1^ of glucan) using Celluclast 1.5 L and Novozym 188. As a result, the authors observed that in assays with the lowest enzyme dosage the period to achieve the maximum glucose concentration was larger than those with higher enzyme dosages where maximum glucose concentration was achieved between 48 h and 72 h of reaction for all solids loadings tested. Additionally, corn stover was hydrolyzed with 30 FPU Celluclast 1.5 L per gram of biomass and 30 IU cellulase from *Aspergillus niger* per gram of biomass for the production of bioethanol. The effect of decreasing enzyme loadings (75%, 50%, 25%, and 10%) was studied. The net glucose amount depended on the enzyme loading and time the sample was taken. All enzyme loadings achieved at least 80% glucose yields after 48 h of hydrolysis [39].

In our study, the hydrolysis of argan pulp using Viscozyme yielded a greater reducing sugar concentration than Celluclast in the same conditions. This can be attributed to the Viscozyme composition. Viscozyme contains a large range of carbohydrases, in addition to cellulase and glucosidase, allowing high hydrolysis of argan pulp, which is composed of cellulose and hemicelluloses.

#### 2.2.2. Effect of Biomass Loading

Figure 1 shows the evolutions of reducing sugar concentrations over time for three different biomass loadings (2%*_w_*_/*v*_, 5%*_w_*_/*v*_, and 10%*_w_*_/*v*_) at different enzyme concentrations. Basically, it can be noted that, in general, the higher amount of hydrolyzed substrate, the higher the amount of reducing sugars, as expected. Surprisingly, the conversion yield increased rapidly when the substrate loading was increased from 2% to 5% (Figure 1a–d). Thus, the higher reducing sugar content was shifted from 321.01 ± 0.5 to 501.20 ± 0.36 mg/g DW and from 376.24 ± 0.14 to 521.46 ± 0.32 mg/g DW after 48 h for assays with Celluclast and Viscozyme, respectively. Argan pulp with a higher enzyme loading demonstrated a higher production, which reached 700.36 ± 0.8 and 723.36 ± 0.22 mg/g DW for Celluclast and Viscozyme, respectively, at a biomass loading of 10%*_w_*_/*v*_ (Figure 1e,f). This means that the hydrolysis rate was nearly proportional to the initial substrate loading. Another way to increase bioethanol production through enzymatic hydrolysis is to enhance the operability of hydrolysis by using higher substrate concentrations, which influences the rate of hydrolysis in order to augment glucose yields in hydrolysates [40,41,42]. Additionally, Qiu, et al. [43] investigated the effect of substrate loading during enzymatic hydrolysis using a wheat straw pretreated with phosphoric acid and hydrogen peroxide. Four substrate loadings (2%*_w_*_/*v*,_ 10%*_w_*_/*v*_, 15%*_w_*_/*v*_, and 20%*_w_*_/*v*_) were tested, and it was pointed out that the highest substrate consistency led to the highest glucose concentration in the final hydrolysate.

Finally, contrary to expectations that predicted an increase in reducing sugar concentration coupled with a decrease in yield when substrate content was increased, it was found that the lowest substrate loading investigated (2%*_w_*_/*v*_) led simultaneously to the lowest conversion yield (38% ± 3%) and the lowest concentration of reducing sugars (197.86 ± 0.30 mg/g DW). Additionally, for argan pulp in the absence of enzymes (control), there was not a significant difference on the production of reducing sugars even after increasing the pulp concentration, hence the need for enzymatic hydrolysis. Thus, by increasing pretreated substrate and enzyme loadings, higher concentrations of fermentable sugar are available and, consequently, higher ethanol concentrations can be achieved. In this study, in presence of the higher concentration of Celluclast and Viscozyme, we obtained a conversion yield of 91% and 88%, respectively, based on the concentration of total sugars before hydrolysis. Similarly, pomegranate peels have been used for bioethanol production using a cellulase enzyme where glucose conversion resulted in a 95% yield [44]. Additionally, the production of bioethanol was studied from hornbeam residues using commercial enzyme cellulase (Cellic Ctec2) where glucose yield was investigated by adjusting three parameters, including severity factor of pretreatment, total solids of enzymatic hydrolysis, and enzyme loading. The optimization of those parameters resulted in a 68% sugar yield, which corresponds to ethanol production of around 250 L/ton of dry raw material [45]. Additionally, a comparative dilute acid pretreatment study was investigated to improve the saccharification of argan pulp using a composite value of total sugars, and reducing sugars as the response value [24]. The results showed that dilute sulfuric acid at T < 160 °C with a high solids loading (40%) led to the highest total and reducing sugar yield at 171.46 mg/mL and 54.83 mg/mL, respectively.

### 2.3. Bioethanol Production

To produce bioethanol, the enzymatic hydrolysis process must provide, as much as possible, a hydrolysate highly concentrated in fermentable sugars. The hydrolysates obtained from the enzymatic hydrolysis of 10%*_w_*_/*v*_ substrate loading presented a satisfactory reducing sugar concentration of 700.36 ± 0.8 and 723.36 ± 0.22 mg/g DW for Celluclast and Viscozyme assays, respectively. Therefore, they were used as a substrate for their bioconversion into ethanol by *Saccharomyces cerevisiae* ATCC 7754. In these assays, the initial sugar concentrations in the bioreactor were measured at about 36.01 g/L and 32.05 g/L for argan pulp in presence of Viscozyme (30 FBGU/g) and Celluclast (30 U/g), respectively, and 50%*_v_*_/*v*_ of the inoculum (as described in Section 3.5.2). As already stated, the medium containing argan pulp in absence of enzymes (control) represented the same medium as hydrolysate argan pulp, whereas the culture medium was supplemented by the minerals and vitamins nutrients, as described by Kristiansen (1994) [46](see Section 3.5.1), with an absence of pure glucose. However, the glucose medium represented a similar medium with an identical concentration of pure glucose.

**Figure 2:**
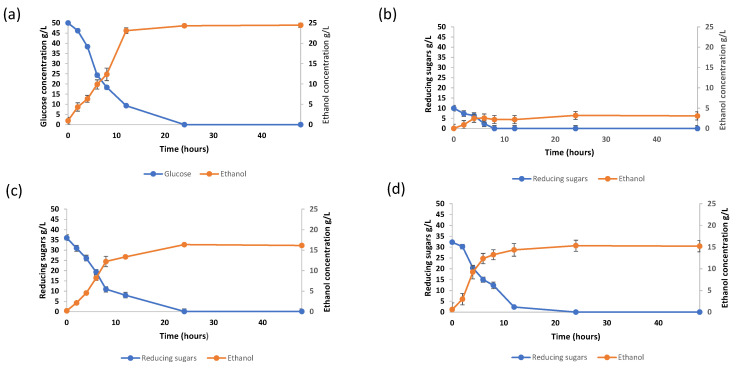
Evolution of ethanol and reducing sugar concentrations (g/L) during fermentation for the four samples studied: (**a**) glucose, (**b**) unhydrolyzed argan pulp, (**c**) argan pulp hydrolyzed using Viscozyme, and (**d**) argan pulp hydrolyzed using Celluclast.

Figure 2 illustrates the evolution of reducing sugar content and ethanol concentrations for the two hydrolyzed argan pulp samples with initial reducing sugar concentrations of 36.06 g/L and 32.25 g/L for Viscozyme treatment (Figure 2c) and Celluclast treatment (Figure 2d), respectively, as well as the unhydrolyzed argan pulp sample (Figure 2b) and the glucose 50 g/L medium control (Figure 2a). Regarding the results of the hydrolyzed samples, the sugars undergo ethanol production in both cases, but the difference lies in the rates of glucose consumption and of ethanol production, and, consequently, in the concentration values compared at the same time point.

In the case of the glucose assay (Figure 2a), the maximum concentration of ethanol was found to be 24.44 ± 0.7 g/L after 24 h, which represented 48.88% of the conversion yield and constituted 97.8% of the theoretical conversion yield (glucose to ethanol). In parallel, the maximum concentrations of ethanol using Celluclast and Viscozyme were 15.21 g/L and 16.14 g/L (Figure 2c,d), respectively, which represented a 47.16% and 44.82% of conversion yield and constituted 94.32% and 89.6% of the theoretical conversion yields according to the Gay-Lussac yield law. In addition, the higher concentration of ethanol was only 2.6 ± 0.2 g/L at the same time for the unhydrolyzed assay, hence the need for enzymatic hydrolysis. Similarly, the production of ethanol in the simultaneous saccharification and fermentation of corn straw using a combination of the two enzymes Celluclast and Viscozyme was studied. The process resulted in 16.98 g/L of ethanol, which represented a 31% yield [47]. Hornbeam residues were also investigated using a commercial cellulose enzyme (Cellic Ctec2). An optimal production process resulted in a 68% sugar yield, which corresponds to an ethanol production of around 250 L/ton of dry raw material [45]. Additionally, vetiver grass, a lignocellulosic-rich material, was also used with cellulolytic enzymes for bioethanol production. The highest sugar contents from enzymatic hydrolysis and bioethanol production were 21 g/L and 6 g/L, respectively [48].

Finally, fermentation of argan pulp pretreated using *Saccharomyces cerevisiae* enzymes led to an alcoholic fermentation with a significant yield of bioconversion (47.16% and 44.82% using Celluclast and Viscozyme pretreated samples, respectively), which seems consistent with the absence of inhibiting during the hydrolysis steps. In our study, we produced 304.2 g and 322.8 g of ethanol per kg of argan pulp using Celluclast and Viscozyme, respectively, which constituted a very significant bioethanol production yield. Similarly, Carob solid waste was used for the production of bioethanol using acid hydrolysis (HCl 1N) and *S. cerevisiae*, where conversion yield was maintained and achieved up to 155 g of ethanol per kg of waste during solid state fermentation [49].

## 3. Materials and Methods

### 3.1. Argan Pulp Biomass Collection and Preparation

The argan pulp used in this study was provided by Nectarome (Marrakech, Morocco) (https://www.nectarome.com/ accessed on 21 January 2019), installed 35 km from Marrakech, Morocco, and taken from an annual feedstock (2019). It was already dried and ground into a fine powder. After that, the biomass collected was stored until use.

### 3.2. Argan Pulp Chemical Characterization

Ash content and dry matter were determined, first, according to the Association of Official Analytical Chemists method [50]. Fat was determined using the Soxhlet method with petroleum ether [50]. Total protein content was determined according to a Bradford assay using bovine serum albumin (BSA) as a standard [51]. Briefly, 50 µL of the protein extract was added to 1.5 mL of the Bradford reagent, after stirring the mixture was incubated at 30 °C for 30 min. The A_595_ was measured. Bovine serum albumin was used as a standard. Sugar contents were estimated spectrophotometrically using the phenol-sulfuric acid method [52]. Reducing sugars were determined using a dinitrosalicylic acid (DNS) reagent according to the method described by Miller [53]. Lignin, cellulose, and hemicellulose were determined according the method described by Van Soest [54] using acid digestible fiber (ADF) and neutral digestible fiber (NDF). Total phenolic compound content was quantified using the Folin–Ciocalteu method with gallic acid as a standard [55]. To determine total phenolics, 10 µL of hydroalcoholic extract was mixed with 2 mL of distilled water and 250 µL the Folin–Ciocalteu reagent. After stirring for 3 min, 500 µL of sodium carbonate (20%*_w_*_/*v*_) was added. The mixture was incubated at 40 °C for 30 min. The A_760_ of the mixture was measured. The results of the total phenolic content were expressed as mg of gallic acid equivalents (GAE) per g of initial dry matter (mg GAE/g DM). The results of each parameter were expressed in percentages (%) in proportion to the dry matter (DM). All these assays were carried out in triplicate.

### 3.3. Enzymes and Other Chemicals

The enzymatic hydrolysis of argan pulp was carried out using the following enzymes:

- Viscozyme L; Viscozyme is a cellulolytic enzyme complex containing a wide range of carbohydrases, including arabinase, cellulase, *β*-glucanase, hemicellulase, and xylanase from *Aspergillus* sp. The activity of Viscozyme L was ≥100 Fungal Beta-Glucanase Units (FBGU)/g.
- Celluclast 1.5 L; Celluclast 1.5 L is a liquid cellulase from *Trichoderma reesei* with an enzyme activity ≥700 units/g.

One unit or 1 FBGU corresponds to 1 µmol glucose released/min.

All chemical reagents, enzymes, standards and solvents were purchased from Sigma-Aldrich (Lyon, France).

### 3.4. Enzymatic Hydrolysis

Enzymatic hydrolysis of argan pulp (particle size ≤ 500 μm) was performed in a 250 mL Radleys reactor (Carousel 6 Plus Reaction Station) with a rotational mixing speed of 200 rpm. Argan pulp powder was weighed based on the treatment 2%*_w_*_/*v*_, 5%*_w_*_/*v*_, and 10%*_w_*_/*v*_. These conditions were chosen because the homogeneity of our mixture could be maintained, which was not validated by further increasing the substrate loads. Enzymatic saccharification was carried out in 200 mL of distilled water using Celluclast at 50 °C and pH 5, and Viscozyme at 44 °C and pH 4.5. Different enzyme loading concentrations were tested: Celluclast at 15 U/g, 30 U/g, and 45 U/g of substrate, and Viscozyme at 15 FBGU/g, 30 FBGU/g, and 45 FBGU/g of substrate. The enzymatic hydrolysis was performed for 48 h and samples were collected for analysis at 0 h, 2 h, 4 h, 6 h, 8 h, 24 h and 48 h. The supernatant was stopped by immediate boiling in a water bath at 95 °C for 10 min. After that, samples were centrifuged (1300× *g*, 10 min) and used for the determination of reducing sugars as described above in Section 3.2. A control was carried out in the absence of enzymes under the same conditions.

### 3.5. Fermentation of Pretreated Argan Pulp

#### 3.5.1. Inoculum Preparation

With regard to fermentation, *Saccharomyces cerevisiae* ATCC 7754 was used. The strain was maintained onto YM agar-agar (Yeast Medium, Difco 0712-01-8) at 28 °C. The strain was then stored at 4 °C and subcultured on a Petri dish for 24 h before being used in the culture vessels [49]. The fermentation itself was then carried out in 500 mL Erlenmeyer flasks with a working volume of 400 mL. The culture medium was supplemented with additional elements as defined by Kristiansen (1994) [46] and incubated at 35 °C at a speed of 150 rpm for 18 h. The biomass growth was followed by measuring A_550_ (Biomate 3S, UV/vis spectrophotometer, Thermo Scientific, Lyon, France), and the dry matter was determined by gravimetry.

#### 3.5.2. Saccharification and Bioreactor Fermentation of Pretreated Argan Pulp

Enzymatic hydrolysis was conducted as described in Section 3.4 using optimal enzyme conditions and a biomass concentration of 10%*_w_*_/*v*_ in a 250 mL Radleys reactor with a working volume of 200 mL. This higher substrate load was made possible by the improved mixing conditions and was used to maintain the reaction rate and also optimize the production of reducing sugars. Simultaneously, the fermentation step was carried out in a 500 mL Erlenmeyer flask with a working volume of 400 mL. The hydrolysate obtained after the enzymatic hydrolysis was transferred to the bioreactor and then autoclaved at 121 °C for 20 min. The system used involved a set of two identical bioreactors (500 mL) that could be operated in different experimental conditions at the same time (INFORS HT, Bottmingen, Switzerland). The working volume was 400 mL. Each bioreactor was equipped with a pH sensor, a temperature controller, a probe measuring the dissolved oxygen (pO_2_), an aeration system, and a magnetic stirrer. Two-hundred mL of inoculum with an initial A_550_ of 2 was added (50%*_v_*_/*v*_). In the bioreactor, the other parameters were a temperature 30 °C, stirring rate of 100 rpm, pH regulated at 6 by KOH (5 mol/L), and/or an H_2_SO_4_ (4 mol/L) addition under anaerobic condition. The glucose batch culture was used as a control with the same bioreactor and inoculum as an argan pulp batch culture with a complete medium (Section 3.5.1). The culture parameters were a glucose initial concentration of 10%*_w_*_/*v*_, a temperature of 30 °C, stirring rate of 100 rpm, pH regulated at 6 by KOH (5 mol/L) and/or an H_2_SO_4_ (4 mol/L) addition. The kinetics of the fermentation were monitored for 48 h with 2 mL samples taken at 0 h, 2 h, 4 h, 8 h, 12 h, 24 h, and 48 h for reducing sugar measurement (as described in Section 3.2) and bioethanol quantification.

### 3.6. Bioethanol Quantification

Bioethanol was analyzed via a high-performance liquid chromatography (HPLC) device (1260 Infinity Quaternary LC system, Agilent Technology, Palo Alto, CA, USA), equipped with two ionic exclusion columns in series (Rezex ROA 300 × 7.8 mm, Phenomenex, Torrance, CA, USA). The mobile phase involved a solution of 5 mM sulfuric acid at a 0.7 mL/min flowrate. Products detection was done using a refractometer (HP 1100 series, Agilent Technologies, Palo Alto, CA, USA). Before injection, all samples were deproteinized. For that, 125 μL of barium hydroxide solution (0.3 M) and 125 μL of zinc sulfate solution (5%*_w_*_/*v*_) were added to the samples. After centrifugation (Thermo scientific, Lyon, France) for 5 min at 10,000 g and filtration using a 0.2 μm cellulose acetate filter (Chromafil, Steinheim, Germany), the supernatant was analyzed. An ethanol calibration curve was plotted for ethanol quantification of each sample.

### 3.7. Data Analysis and Number of Samples

The results reported for argan pulp hydrolysis and fermentation are the mean values of three for each experiment under the same conditions.

## 4. Conclusions

The feasibility of 2 G bioethanol production using argan pulp as a feedstock was assessed. Enzymatic hydrolysis applied to industrially ground (particle size < 0.5 mm) samples reached interesting values of 91% and 88% saccharification yields based on the concentration of total sugars before hydrolysis using commercial cellulosic extracts Viscozyme (30 FBGU/g) and Celluclast (30 U/g) with a 10%*_w_*_/*v*_ substrate loading condition. At this biomass concentration, higher ethanol concentration could be achieved from subsequent fermentation using *Saccharomyces cerevisiae*, e.g., 16.14 g/L using Viscozyme. As an outcome, this process is a promising area of interest to produce affordable clean energy as well as an interesting avenue to effectively valorize argan byproducts with a production of 322.8 g of ethanol for 1 kg of argan pulp. With regards to the quantity of argan pulp produced each year in Morocco (215 kg/ha/year) we can estimate an industrial production of 69.4 kg of bioethanol/ha/year.

## Figures and Tables

**Table 1:** Content of main chemical constituents of argan pulp values corresponded to mean ± SD (standard deviation) of measurements performed in triplicate.

Composition %*_w_*_/*w*_	Argan Pulp
Dry matter %*_w_*_/*w*_	5.02 ± 0.16
Ash %*_w_*_/*w*_	0.29 ± 0.01
Fat %*_w_*_/*w*_	5.46 ± 0.26
Protein %*_w_*_/*w*_	5.02 ± 0.16
Cellulose %*_w_*_/*w*_	19.35 ± 0.14
* NDF/total fibers %*_w_*_/*w*_	29.26 ± 0.19
** ADF Lignocellulose %*_w_*_/*w*_	22.52 ± 0.05
Hemicellulose %*_w_*_/*w*_	6.75 ± 0.10
Lignin %*_w_*_/*w*_	3.17 ± 0.12
Total sugars (mg/g DW)	789.35 ± 0.11
Reducing sugars (mg/g DW)	120.32 ± 0.05
Total phenolic content (mg EGA/g DW)	76.17 ± 0.12

* NDF: neutral digestible fiber; ** ADF: acid digestible fiber.

## Data Availability

The data presented in this study are available on request from the corresponding author.

## Sample Availability

Samples of the compounds are not available from the authors.
